# Di-CNN: Domain-Knowledge-Informed Convolutional Neural Network for Manufacturing Quality Prediction

**DOI:** 10.3390/s23115313

**Published:** 2023-06-03

**Authors:** Shenghan Guo, Dali Wang, Zhili Feng, Jian Chen, Weihong Guo

**Affiliations:** 1 The School of Manufacturing Systems and Networks, Arizona State University, Mesa, AZ 85212, USA; 2 Oak Ridge National Laboratory, Oak Ridge, TN 37830, USA; wangd@ornl.gov (D.W.); fengz@ornl.gov (Z.F.); chenj2@ornl.gov (J.C.); 3 The Department of Industrial and Systems Engineering, Rutgers, The State University of New Jersey, Piscataway, NJ 08854, USA; wg152@soe.rutgers.edu

**Keywords:** convolutional neural networks, domain-knowledge-informed, resistance spot welding, nondestructive quality evaluation

## Abstract

In manufacturing, convolutional neural networks (CNNs) are widely used on image sensor data for data-driven process monitoring and quality prediction. However, as purely data-driven models, CNNs do not integrate physical measures or practical considerations into the model structure or training procedure. Consequently, CNNs’ prediction accuracy can be limited, and model outputs may be hard to interpret practically. This study aims to leverage manufacturing domain knowledge to improve the accuracy and interpretability of CNNs in quality prediction. A novel CNN model, named Di-CNN, was developed that learns from both design-stage information (such as working condition and operational mode) and real-time sensor data, and adaptively weighs these data sources during model training. It exploits domain knowledge to guide model training, thus improving prediction accuracy and model interpretability. A case study on resistance spot welding, a popular lightweight metal-joining process for automotive manufacturing, compared the performance of (1) a Di-CNN with adaptive weights (the proposed model), (2) a Di-CNN without adaptive weights, and (3) a conventional CNN. The quality prediction results were measured with the mean squared error (MSE) over sixfold cross-validation. Model (1) achieved a mean MSE of 6.8866 and a median MSE of 6.1916, Model (2) achieved 13.6171 and 13.1343, and Model (3) achieved 27.2935 and 25.6117, demonstrating the superior performance of the proposed model.

## 1. Introduction

Deep learning (DL) is attracting increasing attention in the manufacturing community as a useful tool for data-driven process monitoring and quality prediction. As a classical DL model for image processing, convolutional neural networks (CNNs) are commonly applied to image sensing data collected from advanced manufacturing applications. For example, a CNN was integrated with long short-term memory and applied to the infrared (IR) thermal images of a melt pool for porosity detection in laser-based additive manufacturing (AM) [1], and virtual metrology for semiconductors could be automatically achieved with a CNN [2]. The popularity of CNNs is highly related to their superior learning ability, large-scale data processing, and decision-making efficiency. Compared with physics-based process modeling and simulation, e.g., finite-element analysis, CNNs are purely data-driven, convenient to update (by retraining the model with new data), and generalizable to various processes [3]. They also do not pose restrictive assumptions towards the underlying processes, and are, thus, more realistic and easier to implement [4,5]. Compared with traditional machine learning (ML) models, e.g., support vector machines and decision trees, which require manual feature extraction before model training, CNNs demand little data preprocessing and perform end-to-end learning. Their performance can be updated in a nearly online manner when new data are provided [6]. Compared with other DL models, especially those having fully connected layers, CNNs are more computationally efficient due to the reduced connectivity in their model structure [6].

Despite the growing interest and adoption of CNNs, using them for quality prediction is not without challenges. There are two issues requiring urgent solutions. First, CNNs need a large volume of imaging data to train the model. When the number of training images is limited or they are of low-quality due to, e.g., noise or a low resolution, the model training outcome is undesirable and reflected as inaccurate model predictions. Second, CNNs only learn from whatever data are fed to them, but neglect physical or practical meanings from the application. Consequently, predictions may be physically invalid and hard to interpret. How to use the model output to instruct the practice is sometimes unclear.

Having recognized the issues, this study aims to improve the accuracy and interpretability of CNNs in quality prediction by leveraging domain knowledge from manufacturing applications. In data mining, “domain knowledge” is information about data already available either through some other discovery process or from a domain expert [7,8]. “Domain knowledge” here refers to information sources other than the image sensors. In manufacturing settings, this is mainly information from the design and prototyping stage, e.g., working conditions and operational mode. In existing studies, the input of CNNs has mostly been real-time images collected by inline sensors during manufacturing processes. These data are undoubtedly vital resources for quality evaluation, as they directly reflect the dynamics of in-process parts and machine/system status. However, letting CNNs learn solely from sensing data without domain knowledge is not only a waste of useful information, which may convey physical or quality information about a process/part, but also limits the effectiveness of CNNs.

**Figure 1:**
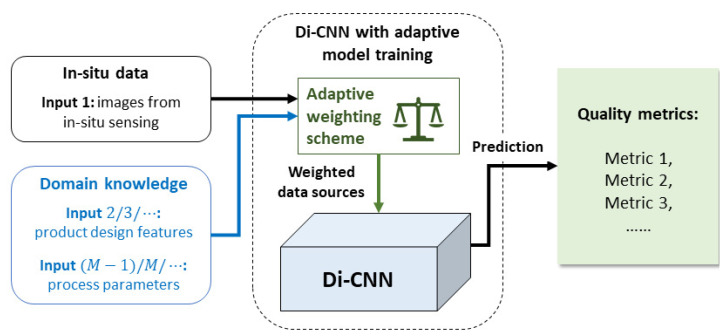
Conceptual overview of proposed method Di-CNN with adaptive input weights.

The objective of this study is to enhance the awareness of CNNs to domain knowledge during manufacturing quality prediction, thus improving their prediction accuracy and interpretability. A novel CNN model was developed named domain-knowledge-informed CNN (Di-CNN) that learns from both design-stage information and real-time image data, and adaptively weighs these data sources during model training (i.e., the adaptivity is in terms of the training data). Figure 1 conceptualizes the model design. Di-CNN explores both design-stage information and part quality/process status, which enhances comprehension and broadens the scope of learning. The adaptive weighting scheme evaluates the relevance of information sources during model training, and increases the weights of those potentially leading to the best performance in quality metric prediction.

The proposed model, Di-CNN, with adaptive weights of data sources, provides a novel solution to the quality prediction challenges associated with CNNs. Di-CNN does not only learn from real-time images during the manufacturing process but also from design-stage information. Augmented input sources enable Di-CNN to be trained with a moderate amount of data, but still predict quality metrics with superior accuracy (see Section 4.2 for details). During model training, Di-CNN generates feedback regarding the most relevant information sources (among the given ones), thereby enhancing the model interpretability (demonstrated in Section 4.3). The most relevant information can be identified as the input with large weights. Future process improvement actions can focus on these input sources or, equivalently, their associated design-stage information. In that sense, Di-CNN can instruct the manufacturing practice, which is not achievable by conventional CNNs.

The rest of this paper is organized as follows. Section 2 reviews the state of the art in related fields. The method development for Di-CNN is elaborated in Section 3. Section 4 provides a case study in resistance spot welding to demonstrate the effectiveness of the proposed model. Section 5 concludes the paper and discusses future research directions.

## 2. Literature Review

In this section, we review the major uses of CNNs in manufacturing and existing works regarding the integration of domain knowledge with DL.

### 2.1. CNNs in Manufacturing

CNNs play a crucial role in advanced manufacturing. One of the major uses of CNNs is image-based process monitoring and quality prediction. Manufacturing processes involving thermal dynamics or rapid part building usually adopt inline image sensors, e.g., pyrometers and high-speed cameras, to acquire real-time images of a part for quality evaluation. Examples include additive manufacturing (AM), resistance spot welding (RSW), and semiconductor manufacturing.

Tian et al. [1], Cui et al. [9], Caggiano et al. [10], and Yan et al. [11] proposed CNN-based approaches toward robust AM quality inspection, i.e., the detection of porosity, cracks, and a lack of fusion. Williams et al. [12] used a 3D CNN to estimate quantitative manufacturing metrics from voxel-based component geometries. Guo et al. [13], and Guo et al. [14] applied CNNs on the thermal images of weld nuggets from RSW for defect detection and nugget dimension evaluation. Ruiz et al. [15] used CNNs to detect fasteners in a real, uncontrolled environment for an aeronautical manufacturing process. Hsu and Liu [16], Lee et al. [17], and Saqlain et al. [18] fed wafer images or the image representation of measurement data to CNN variants for wafer defect detection and system fault diagnosis in semiconductor manufacturing.

In addition to direct quality evaluation, CNNs can be used for feature learning. Weimer et al. [19] developed an automated feature extraction based on CNNs for defect representation. Clustering with particles for object detection was developed on the basis of FastRCNN in Djenouri et al. [20] to identify objects from smart factory images. A two-dimensional CNN underlaid the feature representation scheme in Shi et al. [21] for extracting geometric information from computer-aided design (CAD).

CNNs are also useful in image segmentation and processing. Minnema et al. [22] adopted CNNs for computed tomography (CT) image segmentation to build a 3D surface model in AM. CNNs were demonstrated as an effective and efficient alternative to traditional threshold-based image segmentation. To have efficient support generation in the AM of overhand structures, Huang et al. [23] proposed Surfel-CNN to learn the local topology and status of whether a support is needed at the surfel.

### 2.2. Integrating Domain Knowledge with DL

Criticism against DL is mainly focused on its lack of domain information and interpretability. Review papers indicated the need for enhancing the physics (or domain knowledge) awareness and interpretability of ML/DL models [24,25,26]. This issue is extremely salient in manufacturing fields [27,28]. Targeting this issue, recent studies have attempted to integrate DL models with analytical physics or physical measurements/domain knowledge collected from the field.

There are works that customized the model structure on the basis of the meanings and characteristics of data. For example, with the consideration of nodule heterogeneity in 3D lung nodule images, Zhang and Yoon [29] developed a self-adaptive CNN that incorporated a transverse layer pooling algorithm and a spatial pyramid pooling scheme to adaptively extract equidimensional feature representations from arbitrarily sized images. Wang et al. [30] proposed a deep separable neural network (NN) that applied depthwise separable convolution and dilated convolution in parallel to efficiently handle indistinct tissue characteristics in 3D medical images.

Some studies have pursued the integration of analytical forms of physical/domain knowledge. The physics-Informed NN (PINN) [31] and related works [32,33] are representative efforts to combine the mathematical representation of DL models with analytical physics. The authors used partial differential equations (PDEs) and boundary conditions from physical systems to regularize the optimization objective of deep NNs, ensuring that the trained model obeyed the governing physics of the system. These works were extended to various physical systems, and could be used for system modeling [34] and reconstruction [35].

Unfortunately, many advanced manufacturing processes involve complex physics that is hardly known or mathematically characterized. It is impractical to analytically integrate physics with DL models for these processes. Researchers have attempted to incorporate physical information/domain knowledge from the practice with DL input/output or model structure. True noncontrast images were used in Poirot et al. [36] as a source of domain information, specifically anatomic information, to supervise the training of a CNN for dual-energy CT image processing. A physics-based CNN was developed by Sadoughi and Hu [37] that explicitly considered the rotational speed and fault characteristic frequency as inputs in building convolutional filters. Li et al. [38] proposed WaveletKernelNet in which a convolutional layer was designed to perform continuous wavelet transform, thus guiding the model to learn information of interest from raw data, i.e., the scale and translation parameters in this case. With the guidance of domain knowledge, these DL models achieved improvements in quality prediction performance and model training efficiency.

### 2.3. Summary

Using CNNs on image sensor data for quality prediction has become a trend in manufacturing. Recent studies proposed various CNN-based methods for different applications, but there is no universal solution to ensure robust model performance. A promising direction is leveraging physical and domain knowledge from practice to guide model training and prediction that could potentially improve model training efficiency when data-level restrictions (e.g., small amount, low quality) arise, and enhance model performance. However, current exploration along this track remains preliminary, especially for manufacturing applications. There is no integrated model structure and training algorithm to fulfil the task of domain knowledge incorporation. Developing domain-knowledge-informed CNNs for quality prediction in manufacturing is an imperative demand.

## 3. Method Development

This section develops the Di-CNN model and justifies its validity with theoretical analysis. The classical CNN is first introduced as the base model, followed by the technical details of Di-CNN and its adaptive model training.

### 3.1. Base Model: CNN Regression

**Figure 2:**
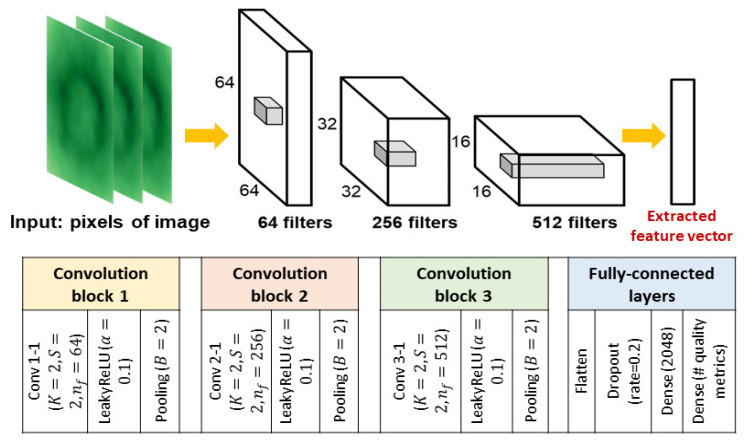
Base CNN model structure used in Section 4 as a benchmark model for performance comparison.

Classical CNNs are neural networks that are applied to image data for classification or regression analysis [39]. Figure 2 shows a CNN model structure that was the base model in this study and one of the benchmarks for performance comparison in the case study (Section 4). The input data are pixel matrices stored in an input layer. Feature extraction is performed on these data with a series of feature extraction layers. Typically, a feature extraction layer consists of *convolutional layers* and a *pooling layer* that are stacked one by one.

A convolutional layer has a set of *filters* (or *kernels*), with each having a small receptive field (side length *K*) and extending through the full depth of the input. For example, the first convolutional layer in Figure 2 has $n_{f}=64$ filters of side length K=2. A filter moves along the row or column of the input for *S* pixels each time until it traverses the two dimensions. *S* is referred to as a *stride*. The output of a convolutional layer may be “padded” with *B* zeros on the border to control its spatial size. According to Nebauer [40], for an input matrix with side length *W*, the output size of a convolutional layer is (W−K+2B)/S+1.

A pooling layer is usually appended to the convolutional layers to complete one feature extraction layer. It downsamples the output of convolutional layers by extracting a single element from each submatrix (of size *P*) inside it. Extraction usually takes the maximum or average of the elements. For example, by letting P=2 in Figure 2, the output volume of convolutional layers would be reduced by 2 in width and length by the pooling layer. To improve the model training performance, a *ReLU layer* may be added between convolutional and pooling layers to form a feature extraction layer [41].

A ReLU layer applies a nonlinear positive-feedback operation to the output of convolution. The function used for this operation is called the *activation function*. Common selections of the activation function include rectified linear units (ReLUs), f(x)=max(0,x), hyperbolic tangents, f(x)=tanh(x), and sigmoid functions, $\sigma \left(x\right)={\left(1+e^{-x}\right)}^{-1}$. ReLU is often preferred due to being able to achieve higher model training efficiency without compromising the generalization accuracy [42].

In classical CNNs, several feature extraction layers are applied to the input data. Eventually, features extracted from pixel matrices are flattened and fed to one or more fully connected layers. Neurons in a fully connected layer connect to all activations in the previous layer [43]. Affine transformation with matrix multiplication is performed to map the features to the predictions of the response. On the basis of the type of prediction, i.e., regression or classification, the activation function in the final layer is selected between ReLU and sigmoid (or softmax), and the model training objective is either minimizing the mean squared error (MSE) or categorical entropy loss.

### 3.2. Di-CNN

Suppose there are *M* relevant data sources (inputs) to the prediction of quality metrics (response). They were obtained with different data acquisition methods and have disparate formats, units, and scales. The major input is real-time images that come as pixel matrices, while domain knowledge inputs are individual design features and build parameters.

Di-CNN fuses multiple inputs at the feature level. A base CNN model (see Figure 2) was adopted for extracting features from the (in situ) image input. The image input may be individual or three-dimensional images, e.g., frames from a thermal video. If more than one image inputs exist, then multiple base CNNs can be adopted, with each extracting features from one image input. For domain-knowledge inputs, if there is a mix of numerical and categorical inputs, dummy coding [44] can be applied to convert the categorical values into numerical values. In the case of domain-knowledge inputs having more complex forms, such as image or time series, a base CNN or base recurrent neural net (RNN) [45] can perform feature extraction from these inputs as they extract features from in situ images. Then, each data source can be expressed with a feature vector $x_{m},m=1,2,...,M$. The length of $x_{m}$ is denoted by $q_{m}$. Among these data sources, some may have higher relevance to part quality and would significantly contribute to performing accurate predictions. This is very likely for certain types of domain knowledge having a strong causal relationship with the quality metrics (the relationship is not explicitly known). To represent such relevance, a weight ($w_{m}$) is given to each data source, i.e., $x_{m}$ is multiplied by the weight to result in $w_{m}x_{m},m=1,2,...,M$. Therefore, weight vector $w={\left[w_{1},w_{2},...,w_{M}\right]}^{T}$ is defined.

**Figure 3:**
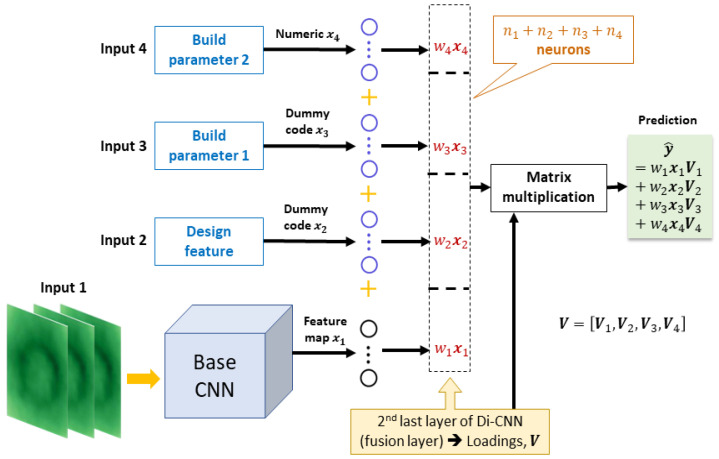
Di-CNN model structure with four input sources.

A *fusion layer* is added after the base CNN. It is a layer of fully connected neurons that amalgamate the weighted features of *M* sources and pass them to the following layers in Di-CNN for quality predictions. Let the numerical response be y, and a vector of length *p* represent *p* numerical quality metrics. Denote the coefficients (or loadings) of neurons [46] in the fusion layer by $V=[V_{1},V_{2},...,V_{M}]$, where $V_{m}$ is a $q_{m}\times p$ matrix, m=1,2,...,M. Di-CNN maps the fusion layer to the response as

(1) $$y\left(w\right)=\sum_{m=1}^{M}w_{m}x_{m}^{T}V_{m}+\epsilon$$

where ϵ is an error satisfying ϵ∼F, and *F* has mean 0 and standard deviation $\sigma^{2}$. The predicted response from Di-CNN is then $\hat{y}\left(w\right)=\sum_{m=1}^{M}w_{m}x_{m}^{T}{\hat{V}}_{m}$. Both y and $\hat{y}$ are functions of w. Figure 3 illustrates the Di-CNN structure with M=4 sources. The model structure of Di-CNN was designed on the basis of practical needs. Its fusion layer is adjustable based on the number of information sources.

### 3.3. Adaptive Weighting Scheme

In practice, the relevance of individual data sources to quality may not be explicitly known, thus posing a challenge in choosing the optimal weight vector to unveil such a varying extent of relevance to achieve accurate prediction. To solve this issue, we propose an adaptive weighting scheme that adaptively optimizes the weight vector during model training. “Adaptive” means that the optimal weights are those minimizing the training-phase validation loss (as measured by MSE), which represents the error between model-fitted quality metrics and the ground truth, for the training data. The proposed adaptive weighting scheme conditionally updates the weights per each training epoch on the fitted neural net coefficients $\hat{V}$.

The weight updating is formulated as an optimization problem. The weights of data sources sum up to 1, i.e., $\sum_{m=1}^{M}w_{m}=1,0\leq w_{m}\leq 1,m=1,2,...,M$. Without loss of generality, we only considered numerical quality metrics, and let Di-CNN adopt MSE as its objective function in model training. Correspondingly, we took MSE as the objective in weight optimization. Let $L\left(w,X,Y,\hat{V}\right):=\bar{MSE}\left(w,X,Y,\hat{V}\right)$, and $V_{m}:=x_{m}^{T}{\hat{V}}_{m}$, which is a vector of length *p*. Then, $V=[V_{1},V_{2},...,V_{M}]$ is a p×M matrix, and ${\hat{y}}_{n}\left(w\right)=\sum_{m=1}^{M}V_{m}w_{m}=Vw$. The weight optimization is as follows:

(2) $$\begin{array}{c} \min_{w}L\left(w|X,Y,\hat{V}\right)=\min_{w}\frac{1}{N}\sum_{n=1}^{N}\frac{1}{p}{∥Vw-y_{n}∥}_{2}^{2}\\ s.t.\;1_{M}^{T}w=1;\;0\leq w_{1},w_{2},...,w_{M}\leq 1\; \end{array}$$

where ${∥\cdot ∥}_{2}$ is a Euclidean norm ($L_{2}$-norm), and $1_{M}$ is a vector of *M* norms.

*L* is a function of $w,X,Y,\hat{V}$ that can be optimized with respect to w given $\left(X,Y,\hat{V}\right)$ to fit Di-CNN coefficients. Alternatively, it can be optimized with respect to $\hat{V}$ given (w,X,Y) to search for the best weights for the training data. The model training of Di-CNN with adaptive weight updating formulates an optimization pair, i.e., $L(\hat{V}|w,X,Y)$ and $L(w|X,Y,\hat{V})$ per epoch (see Algorithm 1). The following three subsections analytically explain the superiority of the adaptive weighting scheme over the conventional training of CNN regression.
**Algorithm 1** Algorithm for Di-CNN training with adaptive weights.Initialize the weight vector w with $0\leq w_{m}\leq 1,m=1,2,...,M$.Extract features from the training data, X (details not shown here).Declare Di-CNN model $model_{0}$.**for** *i* in $1:N_{epoch}$ **do**    Train $model_{0}$ with extracted features X and labels Y for the *i*th epoch.    Minimize $L(\hat{V}|w,X,Y)$ to estimate Di-CNN coefficients.    Take the fitted fusion layer coefficients $\hat{V}$.    Use a nonlinear programming solver to find $w^{\prime }$ that minimizes $L(w^{\prime }|X,Y,\hat{V})$.    **if** $L\left(w^{\prime }|X,Y,\hat{V}\right)<L\left(w|X,Y,\hat{V}\right)$ **then**        let $w=w^{\prime }$.    **end if****end for**Obtain trained model $model_{1}=model_{0}$.Obtain optimized weight vector $w^{*}=w$.

Di-CNN is trained by adaptively updating w in each epoch, such that the model mainly learns from sources that are highly relevant to part quality. Compared with conventional CNNs, Di-CNN is superior in its integration (“concatenation” in terms of the model structure) of both in situ images and design-stage domain knowledge from a manufacturing process, and the inference of the most relevant input sources. Because of this novel model design, the Di-CNN with adaptive weights has better prediction accuracy than that of conventional CNNs (of the same model structure) and interpretable learning outcomes (i.e., the relevance of information sources obtained from Di-CNN model training).

#### 3.3.1. Solving for Optimal Weights

The formulation of Equation (2) is essentially *least squares with linear constraints* [47]. However, it is not the standard least squares with linear equality constraints (LSLEC) [48] due to the presence of inequality $0\leq w_{1},w_{2},...,w_{M}\leq 1$. When *M* is small, slack variables [49], $s={\left[s_{1},s_{2},...,s_{M}\right]}^{T}$, can be used to analytically solve Equation (2). Equation (2) can be reformulated as an LSLEC problem:

(3) $$\begin{array}{c} \min_{w}L\left(w|X,Y,\hat{V}\right)=\min_{w}\frac{1}{N}\sum_{n=1}^{N}\frac{1}{p}{∥Vw-y_{n}∥}_{2}^{2}\\ s.t.\;1_{M}^{T}w=1;w+s=1_{M};\;\\ \;w_{1},w_{2},...,w_{M},s_{1},s_{2},...,s_{M}\geq 0\; \end{array}$$

The analytical solution for w is then derived via a pivot operation [49]. In the case of a large *M*, it would be difficult to derive an analytical solution to Equation (2). We recommend using nonlinear programming solvers, e.g., Sequential Quadratic Programming [50], to numerically solve Equation (2).

The adaptive weighting scheme was designed to optimize the weights given the fitted Di-CNN coefficients. In that sense, optimality is achieved for each epoch rather than globally. Since the weight updating is conditional on the Di-CNN coefficients, the convergence of a stochastic optimization algorithm for model fitting, e.g., stochastic gradient descent [51] or ADAM [52], is highly influential to global optimality. Stochastic optimization algorithms do not always converge in DL model training [53]. In that case, the optimality of weights may also be compromised. On the other hand, if the model training algorithm converges well, the weights tend to stabilize upon the end of model training and reveal the relevance of individual data sources to the quality.

#### 3.3.2. Effect on Sourcewise Correlation

The domain knowledge and real-time image data given to Di-CNN are from the same application. Despite the disparate forms, they are likely to share the same semantics [54] and thus be correlated to some extent. There can also be correlations between different sources of domain knowledge. For example, in RSW, the number of metal sheets to be joined is a design feature, and the current intensity is a build parameter. They are two separate information sources, but the former can be correlated with the latter because a higher current intensity is usually required to join more sheets. Such correlations among various data sources are sourcewise correlations in this context. The presence of a sourcewise correlation is common in multisensory, multichannel data analysis since the data describe the same process from different perspectives. This may distort the actual impact of individual data sources on DL prediction.

The proposed adaptive weighting scheme has a desirable effect on sourcewise correlation. By multiplying the original data sources by $w^{*}$, the initial sourcewise correlations are replaced by weighted correlations that are not larger than the initial ones. Upon optimality, no severe correlations exist among the weighted data sources, so the fitted Di-CNN coefficients are less distorted compared to classical CNNs trained with the same data. This property is proven as follows. Suppose the initial correlations among the *M* data sources form the following matrix:

(4) $$Corr\left(X\right)=[Corr\left(x_{mi},x_{kj}\right)],$$

where $i=1,2,...,q_{m};j=1,2,...,q_{k};m\neq k;m,k=1,2,...,M$, $x_{mi}$ is the *i*th variable in $x_{m}$. $x_{kj}$ is the *j*th variable in $x_{k}$, and Corr(·) is Pearson’s correlation coefficient. By multiplying the data sources with their respective weights, $w_{m}^{*},m=1,2,...,M$, the correlation coefficients are as follows:

(5) $$\begin{array}{c} Corr\left(w_{m}^{*}x_{mi},w_{k}^{*}x_{kj}\right)=w_{m}^{*}w_{k}^{*}Corr\left(x_{mi},x_{kj}\right) \end{array}$$

In Section 3.3.1, the optimal weights should minimize $\bar{MSE}$, i.e., the data sources that tend to substantially decrease $\bar{MSE}$ would be given a larger, nonzero weight. Correspondingly, other data sources may have small or even zero weights. We have the following scenarios:

1. $0<w_{m}^{*}\leq 1,0<w_{k}^{*}\leq 1:$
  $w_{m}^{*}w_{k}^{*}|Corr\left(x_{mi},x_{kj}\right)|\leq |Corr\left(x_{mi},x_{kj}\right)|$
2. $w_{m}^{*}=0,0<w_{k}^{*}\leq 1:\;w_{m}^{*}w_{k}^{*}Corr\left(x_{mi},x_{kj}\right)=0$
3. $0<w_{m}^{*}\leq 1,w_{k}^{*}=0:\;w_{m}^{*}w_{k}^{*}Corr\left(x_{mi},x_{kj}\right)=0$
4. $w_{m}^{*}=0,w_{k}^{*}=0:\;w_{m}^{*}w_{k}^{*}Corr\left(x_{mi},x_{kj}\right)=0$

Scenario 1 is the case in which both $x_{m}$ and $x_{k}$ decrease $\bar{MSE}$. The adaptively weighted correlation between individual variables of either data source is no larger than the original one in an absolute sense. Scenarios 2–4 represent cases in which one or neither of the two data sources help in minimizing $\bar{MSE}$. In these cases, the weighted correlation coefficients between any variables in each source are set to zero. Since Di-CNN model coefficients are estimated at each training epoch with the adaptively weighted data sources, their estimated values are adjusted with the weights from the distortion of original sourcewise correlations. The adaptive weighting scheme contributes to more accurate Di-CNN coefficient estimation.

### 3.4. Model Interpretability

“Interpretability” refers to the extent to which ML/DL predictions can be explained [24]. Di-CNN represents domain-knowledge sources as individual inputs. The fusion layer in Di-CNN was designed to add weights $w=[w_{1},w_{2},\ldots ,w_{M}]$ for *M* data sources (in situ images and domain=knowledge inputs) to quantify their relevance, thus creating an interface for interpreting factors influencing the quality.Specifically, Di-CNN generates optimal weights adapted to the training data. The weights are equivalent to the relevance of data sources to the quality.

Representing the relevance of each data source has practical value. A data source exists due to the capability for data collection from the venue. In manufacturing practice, this is equivalent to having sensors and data processing software installed for the venue. The weights in Di-CNN, as they reveal the data source relevance, are evidence or interpretable indicators to guide future sensor installation and data collection from the manufacturing process. More sensing and data processing resources are assigned to those highly relevant venues, thus improving the data availability and model performance of manufacturing quality prediction in future implementation.

## 4. Case Study

This section demonstrates using Di-CNN for nondestructive quality prediction in resistance spot welding (RSW), and compares the prediction performance of Di-CNN with that of the base CNN (Figure 2) and the Di-CNN without adaptive weights.

### 4.1. Data from RSW

RSW is a widely used joining process in automotive manufacturing that exploits the resistance of metal sheets against electronic currents to generate heat and weld multiple metal sheets at their contact point [55]. In this case study, weld size and thickness were considered quality metrics, as they could be correlated to the strength of the weld. Di-CNN was applied to a lab application of RSW for Boron steel, predicting the diameter and thickness of weld nuggets by learning from the domain knowledge and real-time thermal videos of nuggets.

#### 4.1.1. Data Description

In data acquisition, an IR camera was mounted upon the metal sheets to capture the process of weld nugget formulation after the electrodes had been lifted. The collected real-time data were thermal videos for individual weld nuggets, i.e., one video for one nugget. The IR camera had a high frequency of 100 fps, so each video had over 500 frames. The image size was 61×81. Each frame was a thermal image of the weld nugget, with pixel values representing IR radiation from the nugget surface. Physically, the early stage of nugget formulation involves a low surface temperature, and the electrodes could partially hide the nugget before being fully lifted. Hence, the early frames in a video may show no or little of the weld nuggets. After the nugget had been formulated and stabilized, the mark of the nugget was complete in later frames of the video.

We obtained real-time thermal videos for four operational modes corresponding to four datasets of which the information is provided in Table 1. The thermal videos were Input 1 to the proposed Di-CNN. Features extracted from these videos are denoted by $x_{1}$. Associated with each video is the weld condition or, equivalently, the current intensity used to complete a weld nugget. This design parameter is considered a domain-knowledge input (Input 2). Table 2a shows the welding conditions of selected videos from Dataset i. Current intensity is a categorical variable that we needed to convert into a dummy code for analysis. Among all 4 datasets, there were 9 different weld conditions, so the dummy code was a vector of 9 binary 0–1 elements denoted by $x_{2}$. Each single element represents one weld condition. The “1" means “used", and “0" means “not used". For example, if the first type, light current intensity, is used, then the first element is 1, and the rest are 0, i.e., $x_{2}=\left[1,0,...,0\right]$.

Information about the operational mode of a video is also domain knowledge, including the number of sheets (in a stack of 2 or 3 sheets), sheet thickness in mm (the middle layer in a 3-sheet stack is thicker than the top and bottom sheets), and coating condition (no coating, aluminum coating on the middle sheet only, or aluminum coating on all sheets). These variables are decided in the design stage and stay unchanged for an entire dataset, i.e., all videos in a dataset are associated with the same operational mode. The number of sheets and sheet thickness have a correlated pattern, so we may as well only preserve the number of sheets. Then, the domain-knowledge inputs to Di-CNN also contain the coating condition (Input 3, $x_{3}$, dummy code) and number of sheets (Input 4, $x_{4}$, numerical).

To train the Di-CNN, we also needed the quality metrics as responses (or labels). The metrics of strong interest in RSW are weld thickness (mm) and diameter (mm). Data for these metrics were collected from postprocessing destructive testing. Table 2b shows the measurements for five videos for Dataset i, where Dmin and Dmax are the minimal and maximal diameters of the weld, respectively. Each row in Table 2b corresponds to one nugget, implying that all the thermal images in a video have identical measurements of nugget thickness and diameter.

#### 4.1.2. Image Preprocessing

**Figure 4:**
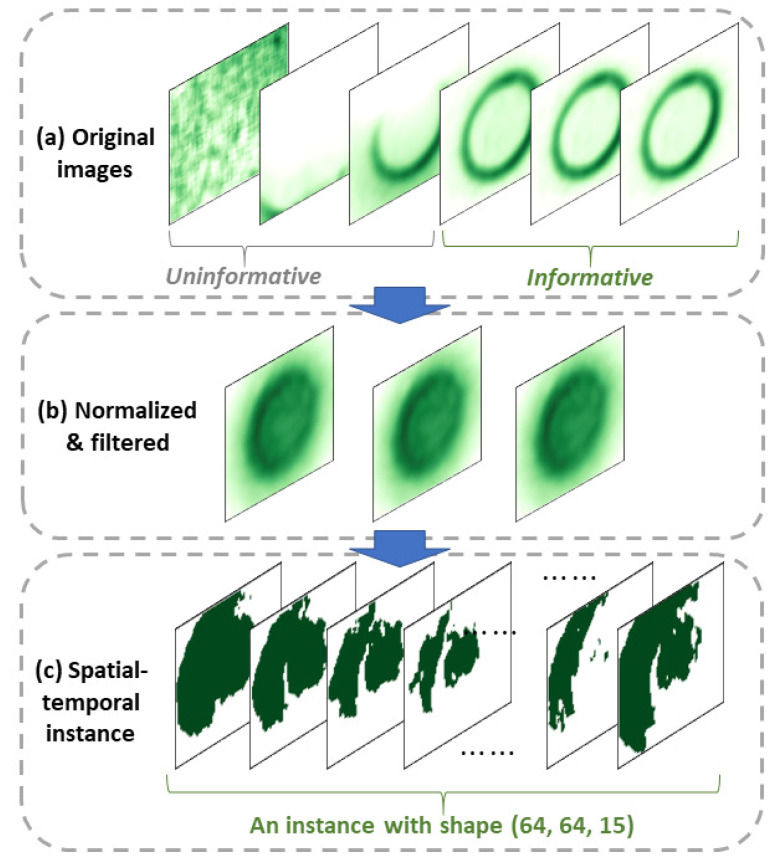
Three-step thermal video processing to build Di-CNN image input ($x_{1}$): (**a**) Original images in a video; (**b**) normalized and filtered frames; (**c**) concatenated frames to be a spatial–temporal instance.

Real-time thermal videos are not directly usable for training a Di-CNN in their initial status. As shown in Figure 4a, the early frames in a video do not have the full mark of a nugget; thus, they are uninformative and unusable for model training. For frames showing a complete nugget, the nugget’s surface pattern is not clear enough due to the low resolution of the IR camera. In addition, there is implicit temporal dependency across the frames in a video. Physically, nugget formulation is a continuous process, so the video, having recorded the process naturally, has a temporal correlation among the frames that must be considered when using the data for model training.

We preprocessed the videos to solve the above issues. First, a raw thermal video was normalized across its frames to reduce the noisy IR radiation incurred during data collection. Then, the 50th percentile of pixel values in the normalized video was used to threshold its frames. Frames whose pixel values were not all equal to or above the threshold were considered uninformative and discarded. Due to the nugget’s stabilization, later video frames were mostly preservable. Figure 4b shows the normalized and filtered images.

Next, *watershed image segmentation* [56] was performed on the preserved frames to characterize the nugget surface pattern. The levels or thresholds of the watershed were the 50th, 60th, 70th, 80th, and 90th percentiles of the pixel values in a preserved frame (i.e., these levels were calculated for each preserved frame). Corresponding to each level, pixels in the frame larger than the level were placed into 1, and the rest were placed into 0, resulting in an image segment. With the 5 levels, 5 image segments were obtained for each preserved frame.

Lastly, to incorporate temporal dependency, image segments of 3 preserved frames with an increment of 5 inbetween were concatenated to form a *spatial–temporal instance*, which is 3-dimensional with the shape of (61, 81, 15). Per the convention of CNNs, we reshaped the images into (64, 64), so a spatial–temporal instance had a shape of (64, 64, 15) (Figure 4c). These became Input 1 to train the Di-CNN.

### 4.2. Performance Evaluation

The Di-CNN was trained with four inputs: (1) spatial–temporal instances, (2) current intensity, (3) coating condition, and (4) number of sheets. The data from RSW had 6116 instances in total after preprocessing. To avoid overfitting and achieve robust performance, sixfold cross-validation (CV) was adopted. The instances were randomly shuffled and divided into 6 folds with equal size. Five out of the six folds were used for model training, and the remaining one fold for prediction (testing). Among the fivefold training data, 90% were taken for model training, and 10% for training-phase model validation. Model training was completed with 500 epochs with no batches used. Three models, namely, (a) the base CNN, (b) the Di-CNN with adaptive weights, and (c) the Di-CNN with fixed weights, were trained and tested with identical data for each CV. For Models b and c, the initial weights were [0.25, 0.25, 0.25, 0.25]. Model b adaptively updated the weights during model training, while Model c used fixed weights.

We summarize the average values and standard deviation of the minimal, mean, median, and maximal MSE losses across CVs in Table 3. The lowest values for these metrics were mainly obtained by Di-CNN with adaptive weights, but the smallest standard deviation for max MSE was achieved by the Di-CNN with fixed weights. The results demonstrate that incorporating domain knowledge into CNNs indeed improves the accuracy of quality prediction. The Di-CNN, even without adaptive weights, had better prediction accuracy and robustness (as represented by the smallest standard deviation) than those of the base CNN. The Di-CNN with adaptive weights, our proposed method, obviously outperformed the two benchmarks in terms of prediction accuracy and robustness.

### 4.3. Interpretation of Optimal Weights

**Figure 5:**
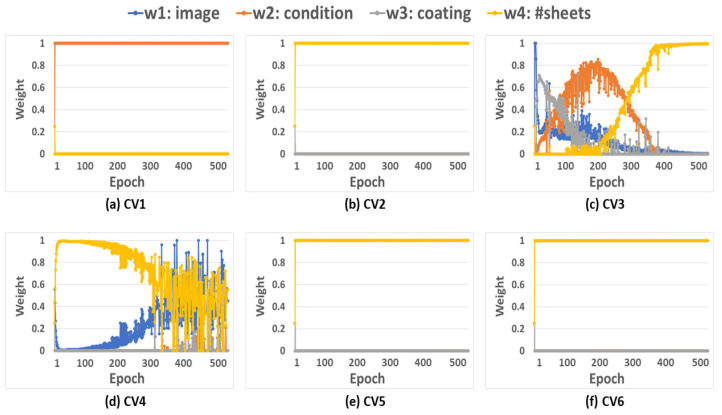
Weight trajectories for sixfold CV. The weights represent the relevance of individual inputs.

Figure 5 displays how the weight of each input evolved during the 500 training epochs in each iteration of the sixfold CV. As model training proceeded, the optimal weights were gradually revealed. When the Di-CNN model stabilized, the weights tended to stay at certain levels. Figure 5c,d show mild fluctuations, while the remaining subplots show stable weights upon training completion. Despite the randomness of the data split in CV, the nonzero weights were $w_{1}$ for the image (Figure 5b–f) and $w_{4}$ for the number of sheets (Figure 5c,d). Inputs 1 and 4 were the most relevant information sources for part quality prediction in this RSW application. Practically, the zero weights for Inputs 2 and 3 did not necessarily mean that these inputs were useless in part-quality prediction, but they were less relevant than Inputs 1 and 4. As an optimization problem, the adaptive weighting scheme finds the most relevant information sources for part-quality prediction, and may heavily weigh the best information source and shrink the rest. In the context of the Di-CNN, a zero weight for input means less or the least relevance, but “less or least” is in a relative rather than an absolute sense. Changing the information sources may alter the weight trajectory if the same input source becomes more relevant than other (new) input sources. Considering the randomness in CV, the training data used in each iteration may also impact the weights’ trajectories. For instance, Figure 5a shows that Input 2 was the most relevant to part quality, which is disparate from the results in Figure 5b–f.

Practically, two factors would affect the inputs’ relevance: the training data, and the causal ground-truth relationship between an input and the part quality. From a data perspective, the included data split and input sources may alter the weight trajectories. From a causal relationship perspective, the ground-truth relationship between inputs and part quality is unknown (and disparate from the concept of relevance). The proposed Di-CNN with adaptive weights mainly infers the relevance of an input source to the part quality from training data. So, the data had a dominant impact on the weight trajectories. Sometimes, the result may differ from the user’s subjective judgment, which is a correction to empirical knowledge. For example, Figure 5 reveals that Inputs 1 and 4, especially Input 4, were more relevant to the weld nugget quality than Inputs 2 and 3 were. This learning outcome implies that the number of sheets was likely the most influential build parameter among domain-knowledge Inputs 2, 3, and 4. The image input (Input 1) was not more relevant to the weld quality prediction than Input 4 was due to the compromised data quality (e.g., low resolution and noisy radiation in the background). The implications from the weight trajectories include: (i) the metal sheet arrangement requires extra attention in RSW practice, and (ii) the image sensor for data collection from this RSW application should be upgraded.

## 5. Conclusions

In this study, a Di-CNN was proposed that adaptively integrates domain knowledge with real-time image data for quality prediction in advanced manufacturing. Di-CNN is informed by data from design and prototyping stage. The adaptive weighting of the information sources was achieved during model training of the Di-CNN. Such a model design improved the prediction performance and interpretability of the Di-CNN. The determined weights in Di-CNN training can be used for root cause identification and decision making. The superiority of Di-CNN was theoretically proven and practically demonstrated with a case study of RSW. The Di-CNN framework is generic and can, thus, include various types of domain knowledge for a wide application in manufacturing. For future extensions, the weights from the Di-CNN may be used to guide data acquisition; more attempts to integrate external information with ML models can be explored.

Indeed, the current Di-CNN has certain limitations that could be resolved in future endeavors. First, it predicts numerical quality metrics, but does not handle categorical ones. Extending the quality prediction to categorical metrics enhances Di-CNN’s applicability. Second, Di-CNN confronts the computing burden in model training as all DL models do. Integrating sparsity into the Di-CNN model structure lessens the model training burden. Third, the current weight updating for Di-CNN is only based on training loss and is not regulated. A more sophisticated weighting scheme may be proposed that considers computational efficiency and a regulated training objective.

## Figures and Tables

**Table 1:** Dataset information for real-time thermal videos.

Dataset	No. of Sheets	Sheet Thickness	Coating Condition	No. of Videos	No. of Frames per Video
(i)	2	1-1	No	25	600–602
(ii)	3	1-2-1	No	38	550–552
(iii)	3	1-2-1	Al (middle)	24	500–504
(iv)	3	1-2-1	Al (all)	22	500–504

**Table 2:** Current intensity and weld quality metrics (in mm) for the selected videos in Dataset i.

Video	(a) Current Intensity	(b) Quality Metrics		
		Thickness	Dmin	Dmax
1	Light	1.899	3.135	3.311
2	Light	1.905	3.135	3.289
3	Minimal	1.871	4.923	4.923
4	Minimal	1.875	4.875	4.945
5	Medium	1.861	5.740	5.762

**Table 3:** Average (standard deviation) of the minimal, mean, median, and maximal MSE losses across CVs.

Model	Min MSE	Mean MSE	Median MSE	Max MSE
Base CNN	6.5055	27.2935	25.6117	78.0820
	(6.9693)	(21.6592)	(20.0323)	(56.2479)
Di-CNN,	**0.4787**	**6.8866**	**6.1916**	**27.6981**
adaptive weights	**(0.8118)**	**(5.2666)**	**(5.2274)**	**(11.0438)**
Di-CNN,	3.4093	13.6171	13.1343	37.1102
fixed weights	(4.3242)	(8.8425)	(9.1238)	(12.7604)

## Data Availability

The datasets generated during and/or analyzed during the current study are available from the corresponding author on reasonable request.
